# The LV map leads to misleading interpretations of coexistence

**DOI:** 10.1073/pnas.2535393123

**Published:** 2026-07-10

**Authors:** Isabell C. Ernst, Jurg W. Spaak

**Affiliations:** ^a^ Institute of Environmental Sciences Landau, Department of Natural and Environmental Sciences, Rheinland-Pfälzische Technische Universität Kaiserslautern-Landau, Landau 76829, Germany

Nguyen et al. (1) proposed the LV map to understand how coexistence changes with time by first fitting a S-map to the time-series, then use partial derivatives of the S-map to construct a LV map, essentially fitting a time-dependent Lotka–Volterra equation to derive a model of the form

$${\frac{1}{N}}_{i}\frac{dN_{i}}{\mathrm{dt}}=\mu_{i}\left(t\right)-\sum_{j}a_{\mathrm{ij}}\left(t\right)N_{j}.$$

Importantly, this acknowledges that interaction coefficients may vary over time, e.g., due to environmental changes or seasonal fluctuations, and hence are in some sense nonlinear. Finally, they apply the structural approach to understand coexistence by asking whether the given interaction matrix $A\left(t\right)$ leads to a feasible equilibrium and how robust this equilibrium is against perturbations. Importantly, applying this structural approach is only meaningful if the underlying model is linear and $A{\left(t\right)}^{-1}\mu \left(t\right)$ is a meaningful quantity at each timepoint (2). While this helps our understanding of Lotka–Volterra models it leads to misleading interpretations of coexistence in any nonlinear community model.

**Fig. 1.**
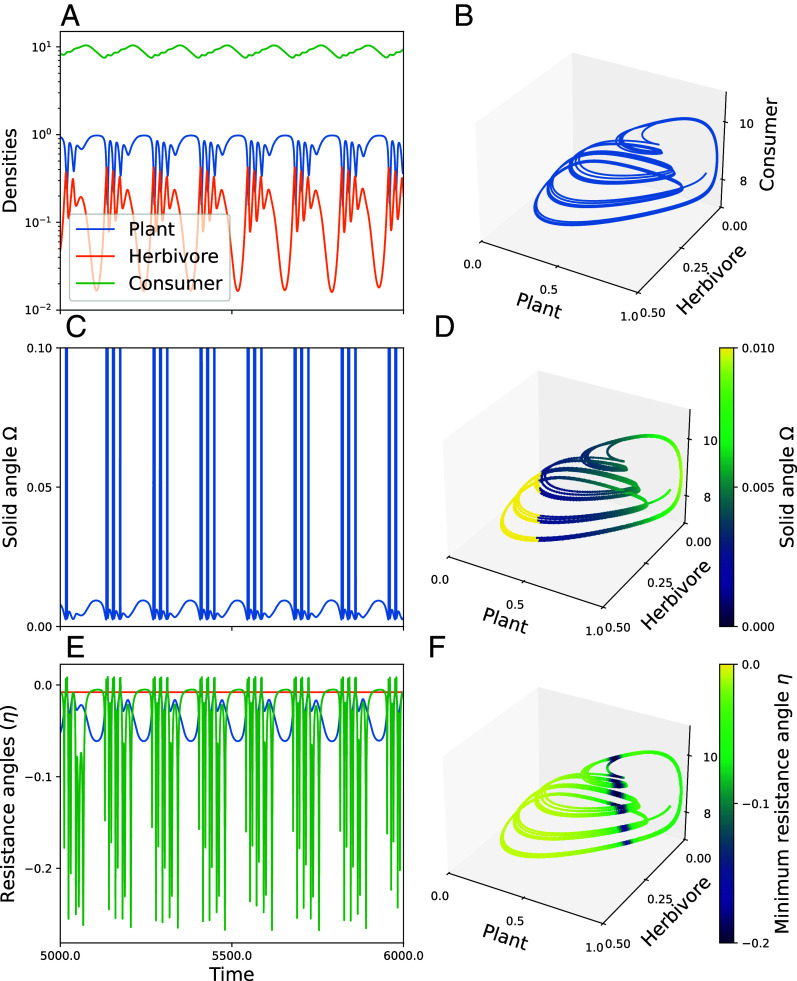
(*A*) The simulations from the food-web lead to chaotic dynamics over time (*B*) shows these densities in the state space. (*C*) The LV map depends on the densities, leading to a solid angle that changes with time suggesting changes of coexistence over time. This is even though the model parameters are constant and species coexist for all times. (*D*) For better visibility we colored the state space accordingly. (*C* and *D*) Ω goes up to 0.5, which we cut for better visibility. (*E*) Similarly, the resistance angle changes over time and is negative, indicating exclusion for most species most of the time, again contrary to the fact that the species actually coexist. (*F*) Similar to *D*, but now colored with the minimum resistance angle. Consequentially, the LV map classifies coexistence incorrectly. Parameters are $a_{ph}=5.0,a_{hc}=0.1,b_{ph}=3,b_{hc}=2,d_{H}=0.4,d_{c}=0.01$.

We apply this idea to a well-known chaotic community model of a three-trophic food-chain governed by Holling type 2 response (Fig. 1 *A* and *B*), given by

$$\frac{1}{P}\frac{\mathrm{dP}}{\mathrm{dt}}=1-P-\frac{a_{\mathrm{ph}}H}{1+b_{\mathrm{ph}}P},$$

$$\frac{1}{H}\frac{\mathrm{dH}}{\mathrm{dt}}=\frac{a_{\mathrm{ph}}P}{1+b_{\mathrm{ph}}P}-\frac{a_{\mathrm{hc}}C}{1+b_{\mathrm{hc}}H}-d_{H},$$

$$\frac{1}{C}\frac{\mathrm{dC}}{\mathrm{dt}}=\frac{a_{\mathrm{hc}}H}{1+b_{\mathrm{hc}}H}-d_{c}.$$

We refer to the original paper for a more detailed explanation of the model (3). Importantly, the model parameters are constant, and species coexist for all times.

Not surprisingly, A(t) from the LV map is time dependent. More importantly, the structural approach misclassifies coexistence in the system (Fig. 1). The structural approach focuses on stable point equilibria and hence cannot be applied to the model we analyzed, nor to the fluctuating data Ngyuen et al. analyzed. The LV map circumvents this by analyzing each time point individually, essentially treating each timepoint as an equilibrium density. Nguyen et al. caution against misinterpreting the results from the LV map. Similar issues occur if the LV map is applied to any nonlinear community model we tested (including higher-order interactions, Rosenzweig-Mac Arthur, and Tilman-Resource competition).

One might argue that this finding is not surprising, our model does not satisfy the linearity assumptions of the LV map, consequently the LV map leads to misleading conclusions. However, the LV map cannot distinguish between nonlinear species interactions and time-dependent linear species interactions. Specifically, Figure 4 in the original paper suggests that the solid angle Ω is low in early spring, implying that coexistence might be most fragile during this time. Yet, this decrease in Ω may simply result from lower densities and nonlinear species interaction, i.e., independent of the coexistence. Generally speaking, empirical applications will include nonlinear species interactions (4, 5), likely leading to misleading conclusions by the LV map, with no way to verify this. Consequently, the reliability of the LV map’s results when using empirical data is questionable.

**Fig. 2.**
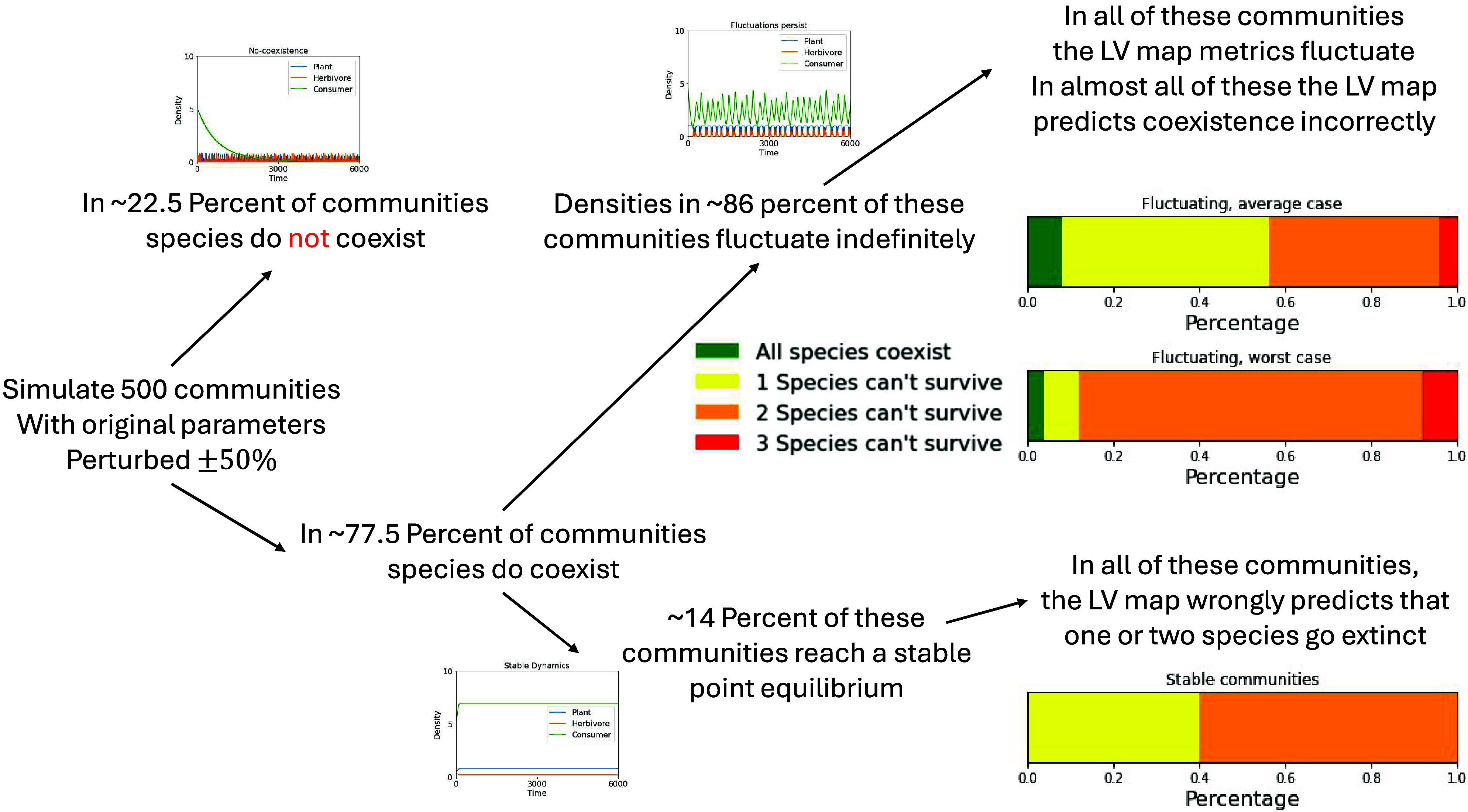
The parameters chosen in Fig. 1 are not a specifically chosen counterexample, but rather the norm. We randomly perturbed the original parameters from Hastings and Powell by 50% and re-analyzed the resulting communities. About 22% of parameter settings did not lead to coexistence, we did not further analyze these communities. In about 14% of the remaining communities all species coexist at stable point equilibrium. Yet, even in these cases, the LV map led to misleading predictions of coexistence. In the remaining 86% of communities the densities fluctuate leading to fluctuating coexistence metrics according to the LV map, despite constant parameter values. Additionally, in almost all communities at least one species was predicted to not coexist at least some time. Finally, even when we take the average over time, only in about 20% of the communities the LV map predicts coexistence correctly. We performed similar analysis for higher-order species interactions and essential resource competition, leading to similar results.

Importantly, the S-map has been invented to handle complex and nonlinear species interactions (6). The LV map fits local linear approximations to these nonlinear interactions. The structural approach treats these local approximations as global approximations, effectively treating the nonlinear species interactions as linear, which generally lead to misleading conclusions as seen in our simulations (~95% in our parameter settings, Fig. 2). Consequentially, the LV map removes one of the main advantages of the S-map.
